# Open Atrial Septectomy on Placental Support: A Novel and Logistic Approach for Hypoplastic Left Heart Syndrome with Intact Atrial Septum

**DOI:** 10.1007/s00246-025-03922-5

**Published:** 2025-06-10

**Authors:** Sameh M. Said, Yasin Essa, Ali H. Mashadi, Geetha Rajendran, Angela Silber, Alexander Mittnacht, Bianca-Nandini Jambhekar, Sangeeta Kumaraswami, Alexandra Linder, Katherine Glover, Melanie Leong, Matthew Pinto, Aalok R. Singh, Kar-Mei Chan, Bhupen Mehta, Supriya Jain, Eric Fethke, Erika B. Rosenzweig

**Affiliations:** 1 Division of Pediatric and Adult Congenital Cardiac Surgery, Maria Fareri Children’s Hospital and Westchester Medical Center, Department of Surgery, New York Medical College, 100 Woods Road, Valhalla, NY 10595 USA; 2 Division of Maternal-Fetal Medicine, Westchester Medical Center Health Physicians Advanced OB/GYN Associates, New York Medical College, Valhalla, NY 10595 USA; 3 Division of Pediatric Cardiac Anesthesiology, Department of Anesthesiology, Westchester Medical Center, New York Medical College, Valhalla, NY 10595 USA; 4 Department of Anesthesiology, Westchester Medical Center, New York Medical College, Valhalla, NY 10595 USA; 5 Division of Pediatric Cardiology, Maria Fareri Children’s Hospital, Boston Children’s Health Physicians, and New York Medical College, Valhalla, NY 10595 USA; 6 Division of Neonatology, Maria Fareri Children’s Hospital, Boston Children’s Health Physicians, and New York Medical College, Valhalla, NY 10595 USA; 7 Division of Pediatric Critical Care, Maria Fareri Children’s Hospital, Boston Children’s Health Physicians, and New York Medical College, Valhalla, NY 10595 USA; 8 Department of Pediatrics, New York Medical College, Valhalla, NY 10595 USA

**Keywords:** EXIT, Open atrial septectomy, Norwood, Hypoplastic left heart syndrome, Intact atrial septum

## Abstract

Hypoplastic left heart syndrome with intact inter-atrial septum continues to be a challenge with high mortality, despite representing only 6% of those with hypoplastic left heart syndrome. No standard management exists for these patients, and centers vary in their approach depending on expertise and available resources. Interventions range from fetal transcatheter balloon atrial septostomy with or without stent placement-to-postnatal transcatheter, surgical, and/or hybrid strategies. In the current report, we present the first successful *Ex* utero, *I*ntrapartum *T*reatment (EXIT)-to-open atrial septectomy-to-rapid stage I Norwood palliation in a neonate with hypoplastic left heart syndrome and intact inter-atrial septum. Creation of unrestrictive atrial communication (open atrial septectomy) was performed at 38 weeks gestation with partial delivery of the fetus who was kept under placental support till the septectomy was completed and without the use of cardiopulmonary bypass. 24-h later, stage I Norwood palliation was completed successfully. In conclusion, Open atrial septectomy under placental support (EXIT) is a logistic approach for hypoplastic left heart syndrome with intact atrial septum. It provides a controlled environment for left atrial decompression and can be followed swiftly with stage I palliation. This rapid sequence first stage palliation may have the potential for producing better results in this challenging subgroup of patients.

## Introduction

Hypoplastic left heart syndrome (HLHS) with restrictive or intact atrial septum (IAS) carries the highest mortality among those with HLHS [1]. Emergency creation of mixing at the atrial level is needed. This can be performed pre- or postnatally. Prenatal interventions are limited to centers with expertise in fetal interventions and are not without risk of fetal demise [2]. Postnatal interventions include transcatheter, surgical, or hybrid approaches. These procedures also can be done during *Ex* utero, *I*ntrapartum *T*reatment (*EXIT*) or immediately after delivery (*IMPACT*, *IM*mediate *P*ostpartum *A*ccess to *C*ardiac *T*herapy) [3]. It is important, however, to appreciate the major difference between those with restrictive vs IAS which has a big impact in terms of the hemodynamic stability of the newborn, and their ability to tolerate transfer to the cardiac catheterization laboratory or the operating room after birth. It is also an important factor in predicting the success of the septostomy in these cases.

In the current report, we present our novel rapid sequence stage I palliation for a neonate with HLHS/IAS that was performed electively at 38 weeks gestation. This included a controlled partial delivery with maintaining the neonate on placental support (*EXIT*) until the open septectomy under inflow occlusion was completed and was followed by stage I modified Norwood palliation in a 24-h period. This represents the first successful report of this management strategy in this challenging subgroup of patients.

## Case Presentation

**Fig. 1:**
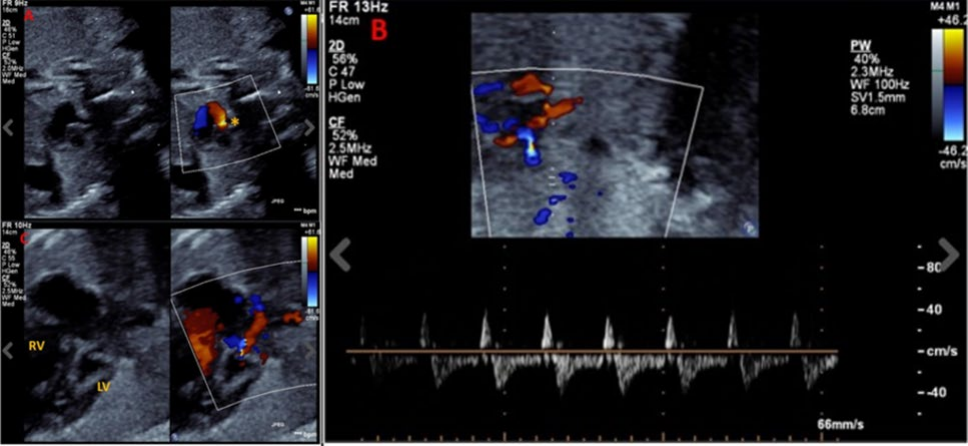
Fetal echocardiogram images at 30 weeks gestation: **A**- a small, restrictive superior atrial communication was seen with left-to-right shunting (asterisk), **B**- pulmonary vein Doppler showing significant reversal, with a forward: reverse VTI ratio of 3:1 and **C**- color compare image showing severely hypoplastic left ventricle and mitral valve. There was significant mitral regurgitation. *RV* right ventricle, *LV* left ventricle

A 29-year-old, gravida 1, para 1 mother was referred to our center for further management of fetal HLHS. Fetal echocardiogram performed at 28 weeks of gestation demonstrated HLHS-IAS with severe mitral valve annular hypoplasia, aortic valve atresia, hypoplastic left ventricle and diminutive ascending aorta. The left atrium was dilated and so were the pulmonary veins, and the atrial septum was thick and muscular with barely any inter-atrial communication (Fig. 1A). Pulmonary venous Doppler showed significant flow reversal during atrial systole and absent early diastolic forward flow, consistent with severe left atrial hypertension (Fig. 1B). There was no decompressing vein or antegrade flow through the left heart. There was significant mitral regurgitation (Fig. 1C). Pregnancy, was otherwise uncomplicated.

Extensive discussion on fetal interventions versus postnatal, favored postnatal intervention, considering the high likelihood of unsuccessful or unsatisfactory result with fetal transcatheter septostomy in cases of IAS and the risk of fetal demise. Continuing follow-up at the time was recommended to detect any fetal hydrops.

### Decision Making and Planning Postnatal Intervention

Multidisciplinary team discussions were ongoing, and considering the thickness of the atrial septum and the lack of a true inter-atrial communication, the decision was made to proceed with EXIT-to-open atrial septectomy.

Alternative management options including compassionate comfort care and transcatheter atrial septal perforation with atrial septal stenting were discussed.

The plan was to admit the patient a few days prior to the planned elective cesarean section date which was determined at 38 weeks of gestation.

### EXIT-to-Open Atrial Septectomy

An EXIT delivery was performed at 38 weeks of gestation. The fetus was partially delivered, and the fetal-placental circulation was maintained. Fetal anesthesia was maintained by the placental circulation and additional intra-muscular anesthesia in the left arm of the fetus. After transthoracic echocardiographic confirmation, a median sternotomy was performed. The pericardium was opened, providing access to the heart and great vessels. External inspection of the heart confirmed the fetal diagnosis of HLHS. A long purse string suture was placed in the free wall of the right atrium. Using bicaval inflow occlusion technique, the superior and inferior venae cavae were temporarily occluded with vascular clamps. The heart was allowed to empty, and the right atrium was entered through an incision within the purse string suture. The atrial septum consisted entirely of muscular wall, with no communication point between the right and left atria. Open atrial septectomy was performed and the heart was de-aired by removing the temporary clamps on the venae cavae. Additional de-airing through the pulmonary artery with a needle was performed. Finally, the purse string was pulled to control the bleeding.

Epicardial echocardiogram confirmed that the septum was wide open, and the newborn was then intubated. Continuous pulse oximetry was reassuring, and the decision was made to deliver the baby by clamping and dividing the umbilical cord.

### Second Operating Room

The neonate was transferred to a separate operating room where an umbilical venous catheter was placed, prostaglandin infusion was initiated, and the chest was temporary closed. The oxygen saturation remained in the high 80 s, and the arterial blood gas and lactate levels were satisfactory while being ventilated on room air. The neonate was then transferred to the cardiac surgical intensive care unit (ICU).

### Follow-up in the ICU

The newborn was admitted to the ICU intubated, paralyzed and mechanically ventilated with a primed ECMO circuit outside the patient’s room, however his hemodynamics were within age-dependent norms.

### Rapid Stage I Modified Norwood

The following day, the neonate was taken to the operating room where stage I palliation was completed successfully. Modified Norwood procedure was performed and the pulmonary blood flow was established via a modified right ventricular-to-pulmonary artery conduit (Sano shunt) which was created by sewing a 5-mm ringed Gore-Tex graft to a 7-mm cryopreserved aortic valve homograft.

### Post-Norwood Course

The postoperative course was uneventful and the chest was closed 48 h later. The neonate was extubated on the fourth postoperative day, however required reintubation for additional two days due to volume overload. After his second extubation, he continued to do well with subsequent weaning of all inotropic support and resumption of enteric feeds.

He was discharged two weeks later and continued to do well during his follow-up.

## Discussion

IAS in the setting of HLHS represents a major risk for mortality and requires immediate intervention postnatally if not during pregnancy [1]. Secondary abnormalities involving the pulmonary vasculature (diffuse pulmonary arterial hypoplasia, arterialization of the pulmonary veins with muscular media and subsequent severe pulmonary hypertension) may occur which further increases the risk of mortality and may make many in-utero or postnatal interventions futile.

Fetal interventions have been utilized by centers with expertise in fetal interventions, however these interventions are associated with a real risk of fetal demise (13% in a recent report) and their success rate is low [2, 3]. In a report from the international fetal cardiac intervention registry which included data from 13 centers, the discharge survival for these patients were quite poor (only 35%) [3].

The mere delivery of the neonate and emergency transfer to the cardiac catheterization lab or the operating room poses significant additional risks. Two potential serious problems may be faced depending on the underlying cardiac anatomy and the variant of the HLHS. As the neonate begins breathing and the lungs expand, there is an increase in pulmonary blood flow resulting in severe pulmonary congestion and pulmonary edema if the obstruction is not rapidly relieved which represents an oxygenation issue. However, there could be also a cardiac output issue in those with mitral atresia where no antegrade flow will be present and this will compromise the newborn’s hemodynamics. This would be the case with the proposed IMPACT strategy in which the newborn is delivered in the operating room with immediate transition to catheter therapy or cardiac surgery after initial intubation and stabilization.

In a two-center report, the IMPACT strategy was the protocol being followed with variation in the way it was performed [4]. In center one, the neonate is delivered in one cardiac catheterization suite and then transferred to a second suite for intervention. In center two, the baby is transported from the maternal center to the cardiac catheterization center. Survival was significantly higher in center one compared to the second one which reflects the need to shorten the time for postnatal intervention as possible. Also, many of these patients had patent foramen ovale and restrictive, rather than IAS. The need for ECMO support for these patients was also high.

In our opinion, it is best to avoid creating an emergency scenario, if possible, in these already high-risk cases. Partial delivery in an elective and controlled fashion and addressing the septum prior to completing the delivery should theoretically achieve the best chances of success for these neonates and this can be only achieved by the EXIT strategy. This, however, requires careful planning and involves multiple teams and requires two operating rooms as we demonstrated in the current case.

A few reports of the EXIT procedure are present in the literature with different management strategies based on the center expertise and the clinical scenario. An EXIT-to-CPB and subsequent open atrial septectomy was performed in a neonate with HLHS (aortic atresia/mitral stenosis) [5]. However, this neonate underwent fetal intervention with atrial septal stenting that was unsatisfactory and premature rupture of the membranes for the mother necessitated an emergency EXIT procedure. After initiation of CPB in that patient, the umbilical cord severely constricted and the baby was delivered and was taken to a second operating room where the stent was removed and open atrial septectomy was performed. The neonate did not survive, and the postoperative course was quite challenging per the authors’ report.

EXIT-to-hybrid atrial septal stenting under echocardiographic guidance was performed at 37 weeks in a neonate with HLHS-IAS, and represents probably the longest survival with this approach [6]. The neonate underwent a hybrid stage I followed by comprehensive second stage at 5 months of age but died due to postoperative complications.

The pediatric cardiac surgeon (SMS) involved in the current report had previous personal experience with EXIT-to-open atrial septectomy under inflow occlusion at a previous institution, which facilitated planning for the current procedure [7]. Although the previously reported EXIT-to-open atrial septectomy was initially successful, the neonate required ECMO support while in the ICU, being weaned later, but developed progressive intraventricular hemorrhage resulting in withdrawal of support.

This is the first reported successful case of an EXIT-to-open atrial septectomy-to-rapid stage I Norwood palliation. In our opinion, several factors contributed to the success of the current case. Careful pre-operative planning is critical. As stated previously, we believe it is important not to create an emergency situation, if possible, considering how sick these patients can be. Fetal interventions are associated with risk of fetal demise and the results are not satisfactory in reaching discharge survival. The IMPACT strategy adds unnecessary risks to the newborn and requires additional time, even in the most prepared settings. Importance should not be placed on the time between delivery-to-cardiac catheterization/surgery, but rather on the delivery-to-atrial septectomy time, making the EXIT strategy more appealing and logical for those with IAS. We also believe adding ECMO or CPB to the equation complicates things and both are associated with high mortality in the literature. So, two theoretically better options remain, which are EXIT-to-hybrid atrial septostomy vs EXIT-to-open atrial septectomy with inflow occlusion, as we described previously and in the current case. Placental support in both scenarios provide the hemodynamic stability needed till septectomy/septostomy is completed. Open atrial septectomy with inflow occlusion is a much faster procedure, albeit, historical. It is also more precise and does not require a lot of manipulation inside the heart of the sick neonate.

We believe that the EXIT procedure to open atrial septectomy is a valuable option for select candidates and especially in those with IAS. It is also important to recognize that this procedure requires extensive coordination between multiple provider groups including obstetrics, pediatric cardiology, neonatology, cardiovascular surgery, and anesthesia (both obstetric and cardiac). Furthermore, this case as well others, reflects the importance of early fetal diagnosis to allow for coordinated care and delivery of these patients in a tertiary care setting that is able to provide for the unique and complex needs of both the neonate and the mother.

## Conclusion

This is the first report of a successful rapid sequence stage I palliation of HLHS-IAS through EXIT-to-open atrial septectomy with inflow occlusion, followed by rapid stage I modified Norwood/Sano procedure. Time is critical in these cases, and we believe this strategy should be strongly considered in similar cases to minimize risks to the sick neonate and provides the maximum hemodynamic stability required to achieve successful atrial mixing.

## Data Availability

No datasets were generated or analysed during the current study.

## Abbreviations

CPB: Cardiopulmonary bypass
ECMO: Extracorporeal membrane oxygenation
EXIT: Ex utero, Intrapartum Treatment
HLHS: Hypoplastic left heart syndrome
IAS: Intact atrial septum
ICU: Intensive care unit
IMPACT: Immediate postpartum access to cardiac therapy
